# Oxidative stress, inflammation, endothelial dysfunction and incidence of type 2 diabetes

**DOI:** 10.1186/s12933-016-0369-6

**Published:** 2016-03-24

**Authors:** Andrew O. Odegaard, David R. Jacobs, Otto A. Sanchez, David C. Goff, Alexander P. Reiner, Myron D. Gross

**Affiliations:** Department of Epidemiology, School of Medicine, University of California-Irvine, Irvine, CA 92697-7550 USA; Division of Epidemiology and Community Health, School of Public Health, University of Minnesota, Minneapolis, USA; Department of Epidemiology, Colorado School of Public Health, University of Colorado, Denver, USA; Department of Epidemiology, School of Public Health, University of Washington, Seattle, USA; Department of Laboratory Medicine and Pathology, University of Minnesota Medical School, Minneapolis, USA

**Keywords:** Young adults, oxidative stress, Inflammation, Endothelial dysfunction, Incidence, Type 2 diabetes

## Abstract

**Background:**

Oxidative stress, inflammation and endothelial dysfunction are interrelated factors in the etiology of cardiovascular disease, but their linkage to type 2 diabetes is less clear. We examined the association of these biomarkers with incident type 2 diabetes (T2D).

**Methods:**

Analysis of 2339 participants in the community-based coronary artery risk development in young adults (CARDIA) study. Participants (age 40.1 ± 3.6 years, 44 % Black, 58 % women) were free of diabetes, and were followed 10 years. Cox regression was used to estimate hazard ratios (HRs) for incident T2D adjusting for the other biomarkers under study, demographic and lifestyle measures, dietary biomarkers, BMI (kg/m^2^) and metabolic syndrome components.

**Results:**

F2-isoprostanes and oxidized LDL (oxidative stress) were positively associated with incident T2D, but the associations were attenuated by adjustment for BMI. C-reactive protein was positively associated with T2D even with full adjustment: HR (95 % CI) = 2.21 (1.26–3.88) for quartile 4 (Q4) v. quartile 1 (Q1). The HR (95 % CI) for T2D for biomarkers of endothelial dysfunction ICAM-1 and E-selectin for Q4 v. Q1 were 1.64 (0.96–2.81) and 1.68 (1.04–2.71) respectively, with full adjustment. Including these two markers in a common risk score incorporating BMI and clinical measures improved the prediction probability of T2D: relative risk for the average person classified up compared to the average person classified down: 1.09, (1.06–1.13), P < 0.0001.

**Conclusions:**

Biomarkers of inflammation and endothelial dysfunction were positively associated with incident T2D. ICAM-1 and E-selectin add to the prediction of T2D beyond a common risk score.

## Background

Oxidative stress, inflammation, and endothelial dysfunction are interrelated components of an etiological network that has been linked to the development of cardiovascular disease (CVD) [1]. This relationship with CVD is exacerbated in those with perturbations in glucose homeostasis such as insulin resistance and diabetes [2, 3]. Oxidative stress, inflammation, and endothelial dysfunction may be ameliorated by a healthy diet and lifestyle and have also been linked to the etiology of insulin resistance and type 2 diabetes (T2D), and thus hypothesized as unifying components tying vascular factors to metabolic and cardiovascular risk [4, 5].

The oxidative stress aspect of this hypothesis is largely born out of evidence from animal and in vitro studies demonstrating that chronic levels of oxidative stress are among the earliest abnormalities in the natural history of insulin resistance and T2D [6]. However, the evidence is less developed in human and population based studies [7–10]. On the other hand the evidence for inflammation in the etiology of T2D is broad. A recent meta-analysis showed a dose–response association between higher levels of inflammatory markers and incidence of T2D [11], although the true mechanism is not well understood and related evidence suggests that elevated levels of inflammation may be intermediates in the pathophysiology of T2D [12]. Related to these concepts, we have previously reported that markers of oxidative stress and inflammation predict the development of insulin resistance and the metabolic syndrome [7, 8, 13]. Lastly, endothelial dysfunction may be a cause or a consequence of oxidative stress and inflammation and is hypothesized to be essential to both the development of T2D and CVD [4]. Indeed, a handful of studies have reported that elevated markers of endothelial dysfunction are associated with increased risk of T2D [14–18].

We are able to study this etiological network of biomarkers in relation to the incidence of T2D in a different context than previous reports. Therefore, we examined the associations of oxidative stress (F2-isoprostanes and oxidized LDL), inflammation (C-reactive protein), and endothelial dysfunction (cellular adhesion molecules) with incidence of T2D. We hypothesized that these biomarkers would positively associate with T2D and add to prediction of incident T2D beyond a common clinical risk score.

## Methods

### Study and data collection

CARDIA is a multicenter, longitudinal investigation of the evolution of cardiovascular disease risk starting in young adulthood [19]. The study began in 1985–1986 with 5115 black and white adults aged 18–30 years from four metropolitan areas (Birmingham, AL; Chicago, IL; Minneapolis, MN; and Oakland, CA). Study participants were sampled to obtain roughly equal numbers of blacks (51.5 %) and whites (48.5 %), men (45.5 %) and women (54.5 %), ages 18–24 years (44.9 %) and 25–30 years (55.1 %), and with a high school education or less (39.7 %) vs more than a high school education (60.3 %). Participants were contacted by telephone every year and examined in person at baseline and 2, 5, 7, 10, 15, 20, and 25 years after baseline. The CARDIA study was approved by the institutional review board of each participating institution, is in compliance with the Declaration of Helsinki, and signed informed consent was obtained from each participant at each examination.

At each clinical examination, participants were asked to present fasting in the morning. Tobacco use, strenuous physical activity, and intake of caffeine, food, and alcohol were proscribed. The examinations followed standardized protocols harmonized over time and included measurements of blood pressure, anthropometrics, phlebotomy, and structured questionnaires on socio-demographics, medical and family history, psychosocial characteristics, and diet, among others.

During each clinic exam blood was drawn from an antecubital vein and after serum separation aliquots were stored at −70 °C until shipped on dry ice to a central laboratory. Details on the collection and storage of plasma samples, laboratory quality-control procedures, and methodology for analysis of plasma triglycerides, HDL cholesterol, LDL cholesterol, and total cholesterol are described elsewhere [20]. Details on the measurement and calibration of glucose and insulin [7], blood pressure measurement [19], and anthropometry (height, weight, waist circumference) have also been previously described [21]. Body mass index (BMI) was computed as weight in kilograms divided by squared height in meters. Physical activity was assessed using an interviewer-administered questionnaire which measured the frequency of 13 different exercise activities during the past 12 months [22]. The total exercise score was in exercise units (a sum across 13 activities of frequency × intensity).

The measurement of biomarkers of oxidative stress, inflammation, endothelial dysfunction and serum carotenoids and tocopherols were made as part of the Young Adult Longitudinal Trends in Antioxidants (YALTA), an ancillary study to CARDIA in the Molecular Epidemiology and Biomarker Research Laboratory (MEBRL) in the University of Minnesota. Specifically, serum oxidized LDL concentrations were measured by competitive ELISA (Mercodia, Uppsala, Sweden) [8]. Plasma F2-isoprostanes were measured with gas chromatography-mass spectrometry [23]. Serum carotenoid and tocopherol measurements were based on high performance liquid chromatography [24]. High-sensitivity ELISA was used to measure serum C-reactive protein (CRP) [13]. Cellular adhesion molecules (CAMs) were measured at the MEBRL. E-selectin (serum) and P-selectin (plasma) levels were measured with ELISA methods from R and D systems Inc (Cat No: DSLE00 and BBE6, respectively.) Soluble intercellular adhesion molecule-1 (ICAM-1) (serum) and vascular cellular adhesion molecule-1 (VCAM-1) (plasma) concentrations were measured by ELISA methods (R and D systems: DY720 for ICAM-1 and DVC00 for VCAM [25]. This assay was not affected by ICAM-1 single nucleotide polymorphism (SNP) rs5491, which occurs primarily in blacks and blocks ICAM-1 detection by some antibodies.

### Assessment of diabetes

Type 2 diabetes was defined as use of diabetes medication (assessed at every visit), a fasting blood glucose level of ≥7 mmol/l (126 mg/dl) (measured at years 15–25), 2 h post-challenge glucose ≥11.1 mmol/l (200 mg/dl) (performed at the year 20, 25 exams), or a HbA1c ≥6.5 % (48 mmol/mol) (assessed at the year 20 and 25 visits). Participants were free of diabetes at year 15 according to medication and fasting glucose criteria examined at all examinations up to and including year 15.

### Statistical analysis

This analysis was restricted to participants in the year 15 exam, the first exam at which all biomarkers of interest were measured in blood collected in a single sitting. We included all participants without a history of diabetes or an adjudicated cardiovascular disease event prior to year 15 [26], who also participated in at least one of the year 20 or 25 exams and had complete data on the spectrum of oxidative stress, inflammation, and endothelial dysfunction biomarkers as well as the covariates included in the analyses. Final sample size was 2339. Of note, only 1982 participants had a measure of VCAM. However, sensitivity analyses for the other biomarkers limiting the overall analytic population to N = 1982 did not display different results, and VCAM had no significant statistical effect in any of the models, thus to improve precision of estimates our analytic sample was N = 2339.

For each biomarker subjects were classified into quartiles based on their levels and participant characteristics were calculated by these quartiles. Proportional hazards (Cox) regression (SAS Proc PHREG) was used to examine the association between the quartiles of each of the biomarkers and incident T2D. We estimated the hazard ratio (HR) and corresponding 95 % confidence interval (CI). Time to event was calculated from the baseline examination (year 15) as 5 or 10 years, namely the timing of the first follow-up examination meeting the criteria for the incident outcome (cases) or censoring at the last CARDIA exam without the incident outcome (censored).

A sequential modeling approach was applied. At later steps of the modeling, covariates were included that may or may not be on hypothetical causal pathways or may be co-determinants of risk. The main model included age (years), study center, race, sex, education (years), cigarette smoking (current, former, never), physical activity (units/week), alcohol consumption (ml/day), family history of diabetes and sum of serum α-carotene, β-carotene, β-cryptoxanthin, and lutein/zeaxanthin (Sum4Carot), and serum α and γ tocopherols. We have reported that Sum4Carot is related to oxidative stress, endothelial dysfunction and inflammatory biomarkers [27], BMI [28], hypertension [29], and diabetes and smoking [24]. Serum carotenoids largely reflect dietary intake of fruit and vegetables [30].

Model 2 included model 1 plus the measures of oxidative stress, inflammation and endothelial dysfunction not included in model 1. Model 3 added BMI to model 2. The last model included a metabolic syndrome (MetS) cluster score, consisting of the average of standardized deviates of the primary components of the MetS (i.e., the average of the z scores of waist circumference, systolic blood pressure, triglycerides, inverse HDL-cholesterol, and fasting glucose) [31]. The score at any age can be computed as: 1/5*[(waist circumference − 77.7)/11.4 − (HDL-cholesterol − 44.6)/10.2 + (triglycerides − 90.5)/52.9 + (SBP − 107.6)/9.2 + (glucose − 88.2)/7.4) [31]. A higher z-score indicates that the components tend to cluster in the higher sections of the distributions, i.e., represent overall higher risk. High scores are almost always the result of high values in two or more components. Consideration of coronary artery calcified plaque (CAC) as a measure of subclinical cardiovascular disease was considered in a sensitivity analysis. Details on the assessment of CAC are reported elsewhere [25]. Presence of CAC was defined as a non-zero Agatston score.

Additionally, we created an endothelial dysfunction index to test for any additive association of the different endothelial dysfunction biomarkers as well as test for an extended dose–response relationship. The hypothesis for this approach was informed by research showing the CAMs work in a “cascade” fashion in atherosclerosis [32], and thus higher levels of multiple CAMs reflect greater endothelial activation/inflammation (dysfunction) than an individual CAM. In an intermediary approach we summed the quartile ranks of the positively associated endothelial dysfunction biomarkers in this analysis with a similar modeling approach. There was no evidence that proportional hazards assumptions were violated for any of the exposures as indicated by the lack of significant interaction between them and time in the models. Tests for trend were performed entering the continuous variable of each biomarker into the models. Effect modification of the associations was considered by BMI, race, and sex.

Lastly, we examined whether these different biomarkers improved the prediction of diabetes relative to a common risk score utilized in research and clinical settings [33]. Due to concerns with net reclassification index (NRI) [34–37], we devised an alternative method. Specifically, to assess the improvement in prediction probability of an alternative risk score (formed by adding variables to a base risk score), we examined the gradient of observed diabetes risk across the reclassification of the predicted probability of the common base risk score. We formed a base risk score as the estimated mean, μB, of a Poisson distribution for predicting incident diabetes that included age, sex, race, family history, body-mass index, fasting glucose level, systolic blood pressure, high-density lipoprotein cholesterol level, and triglyceride level. Each person’s predicted base risk was saved and converted to a base probability using the formula 1 − e^−μB^. We then formed alternative risk scores by adding variables to the base risk score that were positively associated with incident T2D independent of the base risk score covariates in our Cox regression models, updating μB to μA, and forming the alternative probability 1 − e^−μA^. For each participant, we subtracted alternative probability—base probability to get the reclassification probability (alternative probability ≥base probability corresponds to reclassification up, alternative probability <base probability corresponds to reclassification down).

The measure of improvement in prediction probability has two parts: a graphic and a regression coefficient. The graphic displays the observed incident diabetes risk across reclassification probability (downward reclassification and upward reclassification) within quartiles of the base probability. An upward trend within a base risk category from downward reclassification probability—upward reclassification probability indicates improved prediction probability. The regression coefficient arises in a further Poisson regression of incident diabetes (dependent variable) on reclassification probability, adjusting for base probability. The regression coefficient provides the estimated risk difference between the average person who is reclassified up (75th percentile of the reclassification probability) vs the average person who is reclassified down (25th percentile of the reclassification probability distribution) and is expressed as relative risk. A statistically significant regression coefficient indicates the existence of improved prediction probability and the magnitude of the relative risk indicates the extent of improvement in prediction probability. All analyses were conducted with SAS statistical software version 9.2 (SAS Institute, Cary, NC).

## Results

The study sample of 2339 participants was aged 32–47 years old (40.1 ± 3.6), was 56 % white, and 58 % women. Incident diabetes occurred in 222 people (9.5 %). Table 1 displays descriptive measures at year 15 for each of the main variables of interest.

**Table 1:** Participant characteristics according to levels of oxidative stress, inflammation, and endothelial dysfunction: CARDIA Year 15 (2000–2001)

	Q1	Q2	Q3	Q4
Oxidative stress				
F2-Isoprostanes				
N	591	576	579	593
F2-Isoprostanes (ng/mL)	31.9 (5.0)	44.5 (3.4)	58.2 (34.4)	100.2 (36.3)
Age (years)	40.3 (3.6)	39.9 (3.5)	40.3 (3.7)	40.0 (3.8)
Race (% black)	41.0	46.0	42.0	47.0
Sex (% female)	46.0	49.0	57.0	82.0
Education (years)	15.4 (2.5)	15.0 (2.5)	15.2 (2.5)	14.9 (2.3)
BMI (kg/m^2^)	26.2 (4.6)	26.9 (4.9)	28.2 (6.1)	31.7 (7.6)
Smoking (% current)	13.4	19.1	20.4	22.4
Alcohol (% light-moderate)	45.8	47.4	41.1	30.8
Alcohol (% heavy)	7.8	10.9	14.2	14.3
Physical activity^c^	393.2 (281.4)	384.7 (286.2)	336.2 (266.3)	276.1 (239.9)
Carotenoid index	81.9 (46.4)	70.6 (36.5)	61.4 (28.8)	51.2 (26.7)
Oxidized LDL				
N	580	588	598	573
Oxidized LDL (U/L)	48.4 (9.3)	69.7 (4.8)	86.2 (5.5)	113.9 (14.7)
Age (years)	40.1 (3.7)	40.2 (3.6)	40.0 (3.5)	40.1 (3.8)
Race (% black)	38.0	45.0	45.0	48.0
Sex (% female)	69.0	63.0	53.0	47.0
Education (years)	15.4 (2.5)	15.2 (2.5)	15.1 (2.5)	14.8 (2.4)
BMI (kg/m^2^)	26.0 (5.7)	28.1 (6.6)	28.6 (5.9)	30.3 (6.2)
Smoking (% current)	17.5	18.3	20.0	19.3
Alcohol (% light-moderate)	39.1	40.9	43.6	41.4
Alcohol (% heavy)	14.5	11.6	10.8	10.2
Physical activity	366.8 (270.5)	348.9 (281.2)	346.3 (274.4)	328.6 (264.0)
Carotenoid index	67.9 (40.2)	67.8 (38.8)	65.4 (35.0)	64.0 (34.6)
Inflammation				
C-reactive protein				
N	582	602	587	568
C-reactive protein (mg/L)	0.31 (0.12)	0.84 (0.22)	2.23 (0.63)	8.82 (7.24)
Age (years)	39.9 (3.4)	40.2 (3.6)	40.4 (3.7)	39.9 (3.82)
Race (% black)	34.0	37.0	46.0	59.0
Sex (% female)	53.0	50.0	57.0	74.0
Education (years)	15.4 (2.5)	15.3 (2.4)	15.0 (2.6)	14.7 (2.4)
BMI (kg/m^2^)	24.5 (3.7)	26.8 (4.5)	28.8 (5.5)	33.0 (7.5)
Smoking (% current)	13.7	19.4	18.8	23.3
Alcohol (% light-moderate)^a^	45.6	43.1	40.4	35.9
Alcohol (% heavy)^b^	12.1	12.7	12.7	9.5
Physical activity	388.9 (277.8)	372.9 (273.6)	348.1 (274.0)	278.9 (252.9)
Carotenoid index	81.3 (45.5)	69.2 (36.1)	61.9 (32.0)	52.3 (26.4)
Endothelial dysfunction				
ICAM-1				
N	597	589	571	582
ICAM-1 (ng/mL)	111.3 (10.8)	134.9 (5.6)	157.0 (7.2)	204.3 (33.2)
Age (years)	40.2 (3.5)	40.2 (3.6)	40.0 (3.6)	40.1 (3.8)
Race (% black)	29.0	36.0	50.0	62.0
Sex (% female)	64.0	57.0	56.0	55.0
Education (years)	15.8 (2.3)	15.5 (2.5)	15.0 (2.5)	14.2 (2.2)
BMI (kg/m^2^)	25.7 (4.7)	27.3 (5.7)	29.2 (6.0)	30.9 (7.3)
Smoking (% current)	7.9	11.5	18.4	38.3
Alcohol (% light-moderate)	48.5	42.9	36.7	36.7
Alcohol (% heavy)	12.5	9.7	11.6	13.3
Physical activity	388.0 (284.7)	368.0 (270.7)	341.5 (277.4)	290.2 (247.5)
Carotenoid index	80.0 (38.6)	70.5 (36.6)	63.2 (38.3)	50.6 (27.0)
E-selectin				
N	591	580	583	585
E-selectin (ng/mL)	18.4 (4.3)	29.0 (2.5)	38.0 (2.7)	51.7 (7.4)
Age (years)	40.3 (3.6)	40.1 (3.5)	40.1 (3.7)	39.9 (3.8)
Race (% black)	31.0	38.0	49.0	59.0
Sex (% female)	71.0	63.0	53.0	45.0
Education (years)	15.7 (2.4)	15.2 (2.5)	15.1 (2.5)	14.5 (2.3)
BMI (kg/m^2^)	26.1 (5.6)	27.2 (5.4)	29.2 (6.3)	30.6 (6.9)
Smoking (% current)	11.0	17.1	20.5	27.2
Alcohol (% light-moderate)	42.7	42.0	41.8	38.5
Alcohol (% heavy)	11.4	10.4	10.6	14.8
Physical activity	357.1 (274.2)	351.7 (272.0)	348.5 (285.6)	332.6 (258.7)
Carotenoid index	78.1 (40.5)	69.7 (40.1)	60.5 (31.3)	56.0 (31.8)
P-selectin				
N	575	590	592	574
P-selectin (ng/mL)	24.5 (4.0)	32.4 (1.7)	38.8 (2.0)	50.8 (10.7)
Age (years)	40.3 (3.6)	39.9 (3.6)	40.1 (3.8)	40.2 (3.6)
Race (% black)	41.0	44.0	48.0	43.0
Sex (% female)	73.0	65.0	54.0	40.0
Education (years)	15.6 (2.5)	15.0 (2.4)	14.9 (2.4)	14.0 (2.5)
BMI (kg/m^2^)	26.9 (5.7)	27.9 (6.2)	29.2 (6.5)	29.0 (6.5)
Smoking (% current)	11.8	17.3	18.3	28.5
Alcohol (% light-moderate)	40.8	41.8	39.6	43.0
Alcohol (% heavy)	11.6	10.3	12.0	13.4
Physical activity	354.3 (279.1)	333.6 (262.6)	356.7 (277.5)	346.6 (272.6)
	73.0 (38.6)	66.7 (40.5)	62.9 (33.2)	62.1 (34.9)
VCAM				
N	489	497	502	494
VCAM (ng/mL)	352.0 (48.1)	457.0 (25.7)	554.3 (30.4)	741.1 (148.1)
Age (years)	39.8 (3.7)	40.0 (3.7)	40.0 (3.6)	40.7 (3.5)
Race (% black)	67.0	49.0	39.0	22.0
Sex (% female)	61.0	59.0	57.0	56.0
Education (years)	14.7 (2.4)	15.2 (2.5)	15.2 (2.5)	15.5 (2.5)
BMI (kg/m^2^)	29.7 (6.1)	28.9 (6.5)	28.2 (6.5)	26.2 (5.5)
Smoking (% current)	24.6	20.3	16.6	13.6
Alcohol (% light-moderate)	39.0	40.2	39.2	46.8
Alcohol (% heavy)	13.6	11.9	11.9	9.8
Physical activity	323.2 (257.7)	345.7 (286.8)	350.3 (269.3)	371.2 (275.3)
Carotenoid index	63.1 (33.5)	64.4 (32.5)	67.5 (40.2)	70.2 (41.6)

^a^ Alcohol: light-moderate (>0 to ≤7 drinks/week female, >0 to ≤14 drinks/week male)

^b^ Alcohol: heavy (>7 drinks/week female, >14 drinks/week male)

^c^ Physical activity: exercise units

Table 2 displays the hazard ratios for incident T2D according to levels of oxidative stress and inflammation. Relative to the lowest levels (bottom quartile) of F2-isoprostanes a monotonic positive association for risk of T2D in quartiles 2–4 was observed after adjustment for demographic, lifestyle, serum carotenoids and tocopherols, and the other markers of oxidative stress, inflammation, and endothelial dysfunction. Upon adjustment for BMI the association was attenuated. The other marker of oxidative stress in the cohort, oxidized LDL, displayed a threshold association where relative to quartile 1, quartile 3 and 4 displayed a similar magnitude of positive association with incident T2D after adjustment for demographic, lifestyle, and other components of the etiological network. The association was largely attenuated upon adjustment for BMI and the continuous metabolic syndrome cluster score, although it persisted for Q3 v. Q1. There was a strong positive association between higher levels of inflammation (CRP) and incident T2D. The nature of the association was graded in quartiles 2–4 after adjustment for demographics, lifestyle factors and serum carotenoids and tocopherols; although the associations in Q2 and Q3 were positive but varying in strength and the estimates of HR became non-significant with adjustment for the other components of the etiological network. The magnitude of the association was strong for Q4 v. Q1 even after full adjustment for BMI and the MetS cluster score.

**Table 2:** Hazard ratio and 95 % confidence interval of type 2 diabetes according to measures of oxidative stress and inflammation: CARDIA year 15–25 (2000–2001 to 2010–11)

Oxidative stress					
	Q1	Q2	Q3	Q4	*P trend*
F2-isoprostanes					
N cases/N	36/591	45/576	56/579	85/593	
Model 1	1.00	1.12 (0.72–1.75)	1.25 (0.81–1.93)	1.83 (1.18–2.83)	0.005
Model 2	1.00	1.09 (0.70–1.70)	1.28 (0.83–1.98)	1.61 (1.03–2.52)	0.11
Model 3	1.00	1.10 (0.71–1.72)	1.14 (0.73–1.76)	1.22 (0.77–1.93)	0.86
Model 4	1.00	1.19 (0.76–1.86)	1.17 (0.75–1.81)	1.43 (0.90–2.26)	0.71
Oxidized LDL					
N cases/N	31/580	43/588	70/598	78/573	
Model 1	1.00	1.25 (0.79–1.99)	2.06 (1.34–3.17)	1.97 (1.27–3.05)	0.0006
Model 2	1.00	1.29 (0.81–2.05)	2.16 (1.41–3.31)	2.09 (1.37–3.18)	<0.0001
Model 3	1.00	1.03 (0.65–1.65)	1.76 (1.14–2.71)	1.46 (0.94–2.28)	0.03
Model 4	1.00	1.06 (0.66–1.70)	1.62 (1.05–2.51)	1.31 (0.83–2.04)	0.14
Inflammation					
C-reactive protein					
N cases/N	18/582	44/602	51/587	109/568	
Model 1	1.00	1.88 (1.08–3.28)	1.97 (1.14–3.39)	3.98 (2.36–6.71)	<0.0001
Model 2	1.00	1.83 (1.05–3.18)	1.67 (0.96–2.90)	2.95 (1.73–5.04)	<0.0001
Model 3	1.00	1.63 (0.93–2.84)	1.42 (0.82–2.48)	2.06 (1.17–3.61)	0.09
Model 4	1.00	1.64 (0.94–2.87)	1.41 (0.81–2.46)	2.21 (1.26–3.88)	0.022

Oxidative stress

*Model 1* adjusted for age, sex, race, center, education, smoking, alcohol, physical activity, family history, serum carotenoids and tocopherols, *Model 2* Model 1 + alternative oxidative stress marker, inflammation (C-reactive protein), endothelial dysfunction (CAMs), *Model 3* Model 2 + BMI, *Model 4* Model 3 + Continuous Metabolic Syndrome Score

Inflammation

*Model 1* adjusted for age, sex, race, center, education, smoking, alcohol, physical activity, family history, serum carotenoids and tocopherols, *Model 2* Model 1 + oxidative stress (oxldl, isoprostanes), endothelial dysfunction (CAMS), *Model 3* Model 2 + BMI, *Model 4* Model 3 + continuous metabolic syndrome score, *P Trend* P value for continuous variable

Table 3 displays the hazard ratios for T2D according to levels of cellular adhesion molecules (CAMS), markers of endothelial dysfunction. There was a strong, graded positive association in Q2-Q4 of ICAM-1 relative to Q1 for incident T2D after adjustment for demographic, lifestyle, etiologic network covariates, and BMI. However, adjustment for the MetS cluster score attenuated the significance of the association. The strong, graded positive association between levels of E-selectin and T2D was significant even after full adjustment. On the other hand there was no association between P-selectin and VCAM with incident T2DM.

**Table 3:** Hazard ratio and 95 % confidence interval of type 2 diabetes according to measures of endothelial dysfunction: CARDIA year 15–25 (2000–2001 to 2010–11)

	Q1	Q2	Q3	Q4	*P trend*
ICAM-1					
N cases/N	21/597	41/589	59/571	101/582	
Model 1	1.00	1.73 (1.02–2.93)	2.28 (1.37–3.78)	3.20 (1.94–5.27)	<0.0001
Model 2	1.00	1.47 (0.87–2.51)	1.73 (1.03–2.91)	1.94 (1.14–3.31)	0.11
Model 3	1.00	1.37 (0.81–2.34)	1.56 (0.92–2.62)	1.72 (1.01–2.94)	0.07
Model 4	1.00	1.31 (0.77–2.25)	1.50 (0.89–2.52)	1.64 (0.96–2.81)	0.20
E-selectin					
N cases/N	28/591	32/580	61/583	101/585	
Model 1	1.00	0.99 (0.59–1.65)	1.69 (1.07–2.66)	2.48 (1.60–3.85)	<0.0001
Model 2	1.00	1.00 (0.59–1.68)	1.56 (0.97–2.49)	2.10 (1.31–3.38)	<0.0001
Model 3	1.00	1.01 (0.60–1.69)	1.34 (0.84–2.15)	1.76 (1.09–2.83)	0.0008
Model 4	1.00	0.99 (0.59–1.67)	1.30 (0.81–2.09)	1.68 (1.04–2.71)	0.0016
P-selectin					
N cases/N	41/575	42/590	67/592	70/574	
Model 1	1.00	0.92 (0.59–1.41)	1.38 (0.93–2.06)	1.48 (0.98–2.22)	0.097
Model 2	1.00	0.76 (0.68–1.54)	1.02 (0.68–1.54)	0.93 (0.60–1.43)	0.58
Model 3	1.00	0.78 (0.50–1.22)	0.96 (0.64–1.45)	0.89 (0.58–1.37)	0.49
Model 4	1.00	0.78 (0.50–1.21)	0.93 (0.62–1.41)	0.80 (0.52–1.24)	0.32
VCAM					
N cases/N	58/489	56/497	42/502	30/494	
Model 1	1.00	1.14 (0.79–1.66)	0.86 (0.57–1.29)	0.75 (0.47–1.19)	0.30
Model 2	1.00	1.16 (0.80–1.69)	0.87 (0.58–1.32)	0.71 (0.43–1.15	0.09
Model 3	1.00	1.09 (0.75–1.59)	0.89 (0.59–1.34)	0.78 (0.48–1.29)	0.28
Model 4	1.00	1.04 (0.71–1.52)	0.85 (0.56–1.28)	0.83 (0.51–1.38)	0.31

*Model 1* adjusted for age, sex, race, center, education, smoking, alcohol, physical activity, family history, carotenoids and tocopherols, *Model 2* Model 1 + other endothelial dysfunction markers (CAMs), oxidative stress (oxldl, isoprostanes), inflammation (CRP), *Model 3* Model 2 + BMI, *Model 4* Model 3 + continuous metabolic syndrome score

To test the hypothesis that higher levels of multiple CAMs reflect greater endothelial dysfunction and portend higher risk for T2D than an individual CAM we created an endothelial dysfunction index adding the quartiles of E-selectin and ICAM-1 with a possible rank sum of 2–8, the sum of two representing being in both the lowest quartile of each CAM and eight representing being in the highest quartile of each. The results of this analysis are presented in Table 4. Relative to a rank sum of 2–3, there was no association in scores of 4–5, but a strong, graded positive association for an index score range of 6–8. Including all four CAMs in the index did not change the magnitude of the estimates, but provided less precision (data not presented).

**Table 4:** Hazard ratio and 95 % confidence interval of type 2 diabetes according to endothelial dysfunction index (E-selectin + ICAM-1) CARDIA year 15–25 (2000–2001 to 2010–11)

Rank sum	2–3	4	5	6	7	8	*P trend*
N cases/N	22/568	22/396	24/379	43/387	51/320	60/267	
Model 1	1.00	1.31 (0.72–2.37)	1.37 (0.76–2.46)	2.23 (1.32–3.78)	2.90 (1.72–4.89)	3.76 (2.23–6.34)	<0.0001
Model 2	1.00	1.23 (0.68–2.23)	1.25 (0.70–2.26)	2.03 (1.19–3.44)	2.53 (1.49–4.28)	3.07 (1.79–5.25)	<0.0001
Model 3	1.00	1.13 (0.63–2.06)	1.09 (0.60–1.98)	1.81 (1.06–3.09)	2.00 (1.16–3.45)	2.49 (1.43–4.33)	<0.0001
Model 4	1.00	1.22 (0.67–2.23)	1.04 (0.57–1.90)	1.91 (1.11–3.27)	1.92 (1.11–3.30)	2.34 (1.34–4.09)	0.0002

*Rank sum* combined rank sum of quartiles of E-selectin and ICAM-1(1–4 each, 8 total possible), *Model 1* adjusted for age, sex, race, center, education, smoking, alcohol, physical activity, family history, carotenoids and tocopherols, *Model 2* Model 1 + oxidative stress (oxidized LDL, isoprostanes) and inflammation (CRP), *Model 3* Model 2 + BMI, *Model 4* Model 3 + continuous metabolic syndrome score

**Fig. 1:**
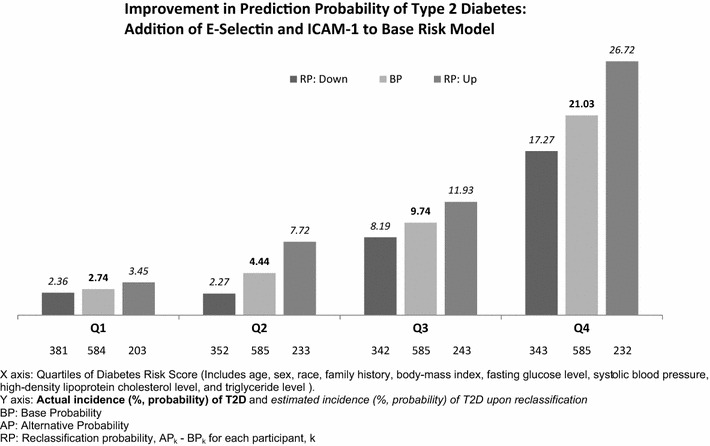
Results from the Improvement in Prediction Probability analysis adding E-selectin and ICAM-1 to a base risk score for T2D: The base risk score was ranked into quartiles (Q1–Q4). For each BP quartile, observed T2D incidence was plotted (*middle bar* in bold) as a reference point without reclassification. For each participant k, reclassification probability < 0 is labeled “Down”. Reclassification probability ≥0 is labeled “Up”. Participants reclassified “down” and “up” are plotted to the *left* and *right* of overall base risk within each BP quartile. The numbers on the *X axis* of the *graph* represent the N reclassified down or up and the overall N of the quartile (N “down” + N “up” = N “base”). The numbers (in italics) above each *bar* represent the estimated probability of incident T2D in participants reclassified down and up. When the probability of incident T2D for reclassification “up” is greater than the probability of incident T2D for reclassification “down”, there is improvement in prediction probability. A formal statistical test used Poisson regression of incident T2D dependent variable) on AP–BP, adjusting for BP. The Relative Risk for the midpoint of those reclassified “up” (75th percentile of AP–BP) vs the midpoint of reclassification “down” (75th percentile of AP–BP) was 1.09, 95 % Confidence interval (1.06–1.13), P < 0.0001

Figure 1 presents the results of the Improvement in Prediction Probability analysis. The graphic displays the results for the addition of E-selectin and ICAM-1 as continuous variables to the base risk score. Within the graphic the observed diabetes incidence was plotted as a reference across quartiles of the base risk score along with downward and upward reclassification probability according to the alternative risk score. The upward trends across categories indicate an improvement in prediction probability over the base probability for that base risk quartile. The formal statistical tests and regression coefficient confirm this as the Relative Risk (RR) for the 75 vs 25th % of the alternative risk score Probability was RR = 1.09, 95 % Confidence interval (1.06–1.13), P < 0.0001. Therefore, there was a relative 9 % improvement in prediction probability with the alternative risk score adding markers of endothelial dysfunction to the base risk score. Alternatively, CRP did not improve prediction probability of diabetes (data not reported).

We carried out a number of sensitivity analyses. There was no evidence that the associations differed for any of the measures by race, sex or BMI. In analyses that accounted for the overall individual factors comprising metabolic syndrome, rather than a continuous score, as well as LDL cholesterol, we observed no material differences in the findings except this approach led to less precise estimates and model fits. Further adjustment for year 15 blood pressure medication also did not affect the results. In another approach we further adjusted for year 15 adiponectin, a potential mediator of these markers and T2D, and this did not impact any of the findings. Since dysglycemia contributes to an environment of oxidative stress, inflammation and endothelial dysfunction we excluded all participants with impaired fasting glucose (IFG) (N = 357) at year 15 and this also didn’t alter the nature of any of the results, neither did excluding glucose from the continuous metabolic risk score. Furthermore, we examined whether the results were dependent on the presence of subclinical cardiovascular disease (CAC) at year 15; neither adjustment for year 15 CAC nor exclusion of 182 participants with any CAC had any material effect on the results.

## Discussion

This population based study, comprised of young to early middle aged black and white adults, showed that biomarkers of an etiological network of oxidative stress, inflammatory, and endothelial dysfunction biomarkers predicted T2D. Biomarkers of oxidative stress, F2-isoprostanes and oxidized LDL, were positively associated with incident T2D, however, adjustment for BMI attenuated the association. We corroborated that inflammation (C-reactive protein) was positively associated with T2D even with full adjustment for related pathways. Lastly, we observed that higher levels of a plasma biomarker of endothelial dysfunction (E-selectin) were strongly associated with T2D even after full adjustment, although another CAM (ICAM-1) was attenuated upon adjustment for metabolic risk factors. An index of these two CAMs revealed an extended dose–response with a greater magnitude of association; whereas the other two CAMs measured (P-selectin and VCAM) were not associated with T2D. Overall, the addition of E-selectin and ICAM-1 improved the prediction of T2D over a common base risk model. There was no evidence any of these results were modified by race, sex, BMI, IFG, adiponectin, or sub-clinical CVD (CAC).

The results from this study related to oxidative stress contribute novel data to the literature as there has been little prospective study of the association between plasma markers of oxidative stress and T2D. In a case-cohort analysis from the ARIC study higher levels of oxidized LDL were positively associated with T2D in a simple model, and in the context of high levels of ICAM-1, but with further adjustment for established risk factors there was no association [18]. Similar results were observed in the Framingham Heart Study for urinary isoprostanes [38]. On the other hand higher urinary isoprostane levels in the IRAS study were inversely associated with T2D [9]. Indeed, urine and plasma measures of isoprostanes are not necessarily equivalent measures [39]. Furthermore, oxidized LDL and isoprostanes represent different aspects of oxidative stress where the former likely represents a local measure in the vasculature and the latter a more general marker of a specific oxidation pathway [40]. These differences may explain the subtleties in the association with T2D in this study.

The results from this analysis related to CRP are confirmatory [11], but the context and population we examined the question in is unique with factors that are likely upstream, downstream and co-determinants. Unlike measures of inflammation only a few studies have examined vascular biomarkers of endothelial dysfunction and risk of T2D. The Nurses’ Health Study observed a strong positive association between higher levels of E-selectin and ICAM-1 with T2D and a non-significant association with VCAM [14]. The WHI had similar findings [16]. The MONICA study observed a similar trend with E-selectin but the ICAM-1 association was only apparent in simple models [15]. The ARIC study only observed a positive association between high levels of ICAM-1 and T2D with high levels of oxidized LDL providing evidence for the hypothesis that endothelial dysfunction needs the presence of oxidative stress to alter T2D risk. We observed a higher incidence rate of T2D with higher levels of ICAM-1 regardless of oxidized LDL level, although the highest rates were in those with the highest levels of oxidized LDL (data not presented).

The results from this analysis are largely consistent with the results from the Nurses’ Health Study and the WHI even with the different design and modeling approach. The population in this analysis was a sample from a community based study, was significantly younger and diverse by race, sex, and socioeconomic position. The results related to the index of levels of E-selectin and ICAM-1 suggest that high levels of both portend a greater risk for T2D than individual consideration of each CAM. Aligned with these results related to vascular biomarkers of endothelial dysfunction and T2D risk are a handful of studies that observed a positive association between other measures of endothelial dysfunction and increased risk of T2D [41–44]. Lastly, despite the evidence of an etiologic role of these markers of oxidative stress, inflammation and endothelial dysfunction in the development of T2D the addition of them to established clinical risk prediction equations did not improve prediction [38, 45, 46]. Highlighting the differences between etiology and prediction. Yet, the endothelial dysfunction biomarkers improved prediction in the current study.

These population studies are evidence in support of vascular dysfunction in the etiology of T2D. But to give this research context it is important to also consider evidence and mechanisms at the cellular level. This literature suggests these factors under study are likely functioning in a cascade or network with pathophysiological consequences [1], but evidence for each has a demonstrated role in the etiology of T2D. Specifically, generalized oxidative stress damages mitochondria and dulls insulin secretion [5]. Oxidized LDL may have an etiologic role by reducing insulin signaling [47], and glucose uptake [48]. In an inflammatory state a cascade of reactions occur that harm vascular reactivity and insulin delivery and lead to insulin resistance [49]. Lastly, clinical and experimental studies suggest that endothelial dysfunction, likely in the capillary and arteriolar endothelium, which are metabolically involved with insulin-sensitive tissues, is the controlling factor for the amount of insulin that effectively reaches the tissues [50]. Thus, there is a strong basic biological precedent for the population level results we and others have observed.

Strengths of this research include the novelty of prospectively examining the association between serum and plasma biomarkers of oxidative stress and endothelial dysfunction with T2D in the context of one another and inflammation. Furthermore, the ability to characterize the metabolic and cardiovascular risk status of the population, and to demonstrate that the observed results were not due to an underlying condition, strengthens the etiological insight this study provides. Additionally, CARDIA has maintained a high participation rate and high quality data collection through rigorous quality-control procedures throughout the study. Thus, the results from this large community-based sample are generalizable to women, men, blacks and whites. Further, the ability to examine this topic in a younger population (age 33–45 years) is a unique contribution to the literature. Limitations include interpretation of the oxidative stress results—the measures of oxidative stress were not correlated with each other, which reflects the complexity of oxidative stress, and emphasizes caution in extrapolating these results to other measures related to oxidative stress. CRP is a non-specific marker of inflammation and other measures such as IL-6, which drive the production of CRP in the liver may be more specific relative to this topic [49]. Endothelial dysfunction was assessed solely by serum/plasma biomarkers and we did not have other measures of vascular physiology. Regardless of measurement, consideration of residual confounding is important in any observational study.

## Conclusions

These findings extend the literature from earlier studies on circulating measures of endothelial dysfunction and risk of T2D, and contribute novel data related to oxidative stress and risk of T2D by assessing these factors in the context of one another and other relevant pathways including a marker of inflammation, metabolic syndrome components and BMI. Overall, these results demonstrate that higher circulating levels of oxidative stress, inflammatory and endothelial dysfunction biomarkers clearly precede the development of T2D.
